# Revolution’s aftermath: population based cross-sectional study to understand the intergeneration mental health and wellbeing following the 2024 student-led uprising

**DOI:** 10.3389/fpsyt.2026.1824190

**Published:** 2026-05-12

**Authors:** Juma Rahman, Bapon Fakhruddin, Md Masudul Haque, Rodrigo Ramalho, Jenni Leppanen, Jubayer Mumin, Mohammed Islam Shakhu, Kazi Mizanur Rahman

**Affiliations:** 1 The University of Auckland, Auckland, New Zealand; 2 Research & Evaluation Office (REO), Auckland, New Zealand; 3 CODATA TG FAIR-DATA, Paris, France; 4 North South University, Dhaka, Bangladesh; 5 Oulun Yliopisto, Oulu, Finland; 6 Karolinska Institutet, Stockholm, Sweden; 7 Federation University NewMed School of Medicine, Brisbane, Queensland, Australia

**Keywords:** Bangladesh, exposure to violence, post-traumatic stress, psychological trauma, revolution

## Abstract

**Background:**

Bangladesh was confronted with a nationwide student uprising in July 2024, that exposed both participants and observers to widespread unrest and traumatic events. To better understand the kind of support the population will need, it is important to understand its immediate impact on mental wellbeing.

**Aim:**

Aim was to examine the prevalence of trauma symptoms among the Bangladeshi general population, aged 15+, within three-months following revolution.

**Methods:**

This cross-sectional survey using the Post Traumatic Stress Disorder (PTSD) Checklist for DSM-5 (PCL-5, five-point Likert scale), was combined with a one-off online workshop to sensitise this population on mental health, trauma, and resilience. Associations between sociodemographic factors and PCL-5 scores were examined with multiple linear regression (ANOVA/ANCOVA). Probable PTSD (PCL-5 ≥ 31) was assessed using log-binomial regression. We estimated Population Attributable Fractions (PAF), Absolute Risk Reduction (ARR) to estimate the proportion of high PTSD attributable to each exposure, and applied min–max normalisation of Likert scales for cross-item comparison.

**Results:**

More than half of the surveyed participants (n=207; mean age 27.6 ± 9.7 years; 72% Gen Z) had clinically suggestive PTSD. This was more common among women (53.7%), and respondents from Chattogram (57.4%) and Khulna (66.7%). Adjusted analyses suggested modestly higher prevalence among Millennials (PR 1.23, 95% CI 0.87-1.74). PAF estimates indicated small contributions from age groups (Millennials +6.5%, GenX/Boomers -3.6%), and gender (men -3.1%). Under hypothetical elimination of exposure, absolute PTSD reduction was greatest among Millennials. Symptom clusters varied: women, and older adults showed consistently higher scores, while Gen Z reported more negative thoughts/feelings.

**Conclusion:**

The study underscores the potential higher prevalence of probable PTSD following large-scale demonstrations and confrontations, and recommends targeted culturally appropriate mental health interventions. Further research involving a representative sample from the population and longitudinal data is recommended to monitor long-term psychological impacts in Bangladesh.

## Background

**Figure 1:**
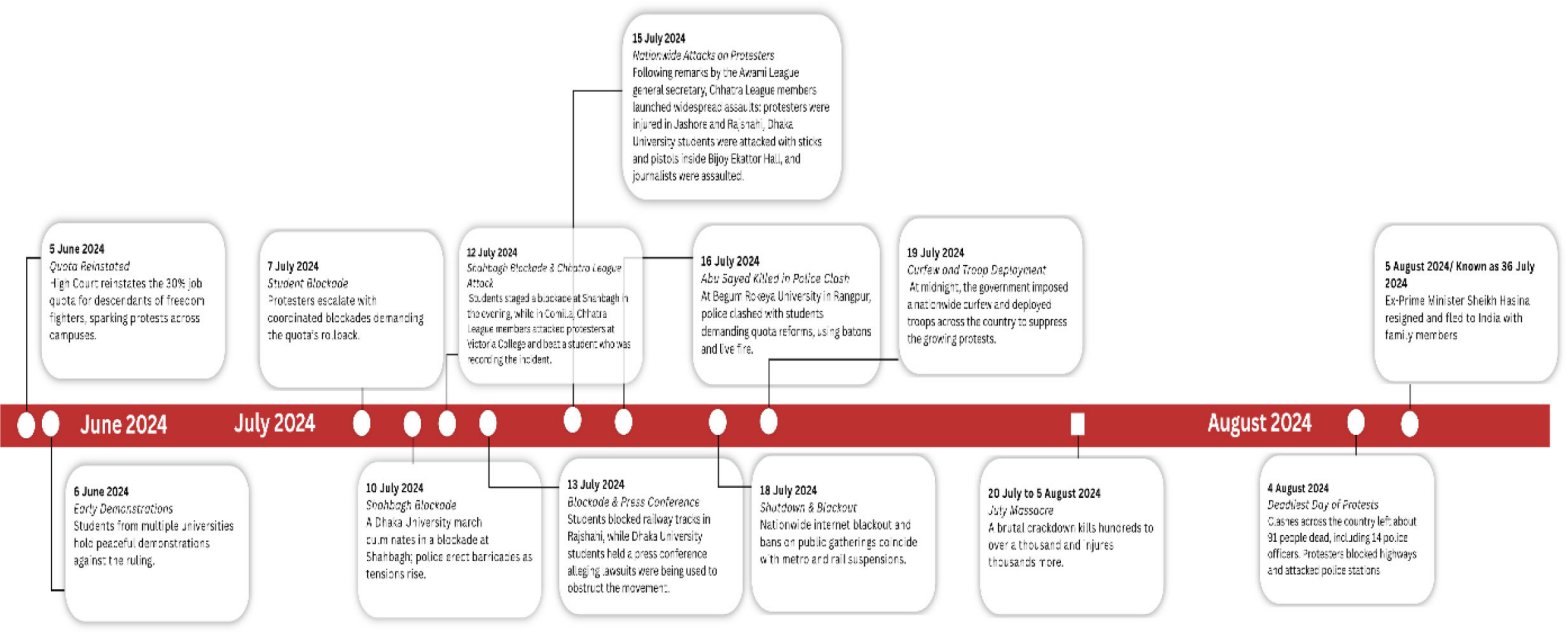
Timeline of the 2024 revolution in Bangladesh (Source: TV and national news papers).

In July 2024, large-scale demonstrations were organised by students from public and private universities and other tertiary-level institutions across Bangladesh, aimed at reforming the government’s public-sector recruitment quota system and advancing broader demands for social equity and justice (1, 2). As the demonstrations escalated, students across the country boycotted academic activities and joined mass protests, and violent confrontations with law enforcement. The movement rapidly transformed into a nationwide uprising (3, 4). During this period, 300 students were killed in one week (3), 1,400 in total in 46 days (5), and Bangladesh experienced a state-imposed internet shutdown (6). In a symbolic act of resistance, students deliberately “extended the calendar” by counting days beyond the conventional end of July, culminating in an ostensible “36th of July” to intensify their demand for the government’s resignation (7), corresponding to 5 August 2024 (**Figure 1**). This collective uprise had a marked impression on population mental health, including among children (8), who were exposed to the unrest through television coverage and social media platforms.

Recent evidence confirms that global social unrest continues to escalate (9–13). Social and political unrests are impacting major urban centres across continents affecting cities, such as Iran, Bahrein, Barcelona, Delhi, Paris, Santiago, Egypt, Turkey, Hong Kong and Nepal. The experience of these political revolutions can offer insights into the situation in Bangladesh, highlighting that political instability and a lack of supportive coping mechanisms can exacerbate trauma and hinder recovery (14, 15). Exposure to mass revolutions can cause collective psychological shifts, influencing societal norms and behaviours (16–19). It results in a spectrum of clinically significant psychological symptoms (14). Individuals may experience persistent and involuntary recollections of the event, often manifesting as intrusive thoughts or flashbacks. Such responses reflect core features of post-traumatic stress and can substantially impair daily functioning and psychosocial wellbeing (20). Trauma classification in psychological research and clinical practice commonly relies on three interrelated criteria: proximity to the event, severity of the incident, and the presence or absence of social support (16, 20). Trauma may also arise from indirect exposure to distressing events through media (8), a phenomenon known as vicarious or secondary trauma (21). Repeated engagement with graphic contents such as news coverage of disasters or political violence can elicit trauma-like symptoms even in individuals not physically present (22). Studies following events for instance the September 11 attacks (23), the Iraq War (24), Israel-Gaza war (25), and aircraft accidents in China (21) have shown that prolonged media exposure, especially exceeding four hours daily, can lead to lasting psychological and physical health effects comparable to direct trauma (26, 27).

Examining the psychological effect of the 2024 student revolution in Bangladesh is crucial not only for the welfare of individuals directly impacted but also for the future stability and resilience of the youth of Bangladesh. This study aimed to evaluate the prevalence and severity of trauma and other psychiatric symptoms, and compared the intensity of trauma across different generations.

## Methods

### Study design and setting

A multiple-method design was employed, comprising a one-off online workshop to sensitise the population on mental health, trauma, and resilience, followed by cross-sectional survey data collection using the Post Traumatic Stress Disorder (PTSD) Checklist for DSM-5 (PCL-5). The recruitment process of the target population is described detailed in **Supplementary Method A1**. Data were collected between October 2024 and January 2025.

The sample size was estimated using the Leslie and Kish formula for estimating a proportion in a cross-sectional study (28, 29). The calculated minimum sample size was 139. Detailed method has been described in the supplementary files (**Supplementary Method A2**). Survey participation was voluntary and anonymous, with data securely stored using Research Electronic Data Capture (REDCap). Ethical approval was obtained from the Public Health Foundation Bangladesh Ethical Review Committee (reference number PHFBD-ERC-SFP21/2024).

### Participants

**Figure 2:**
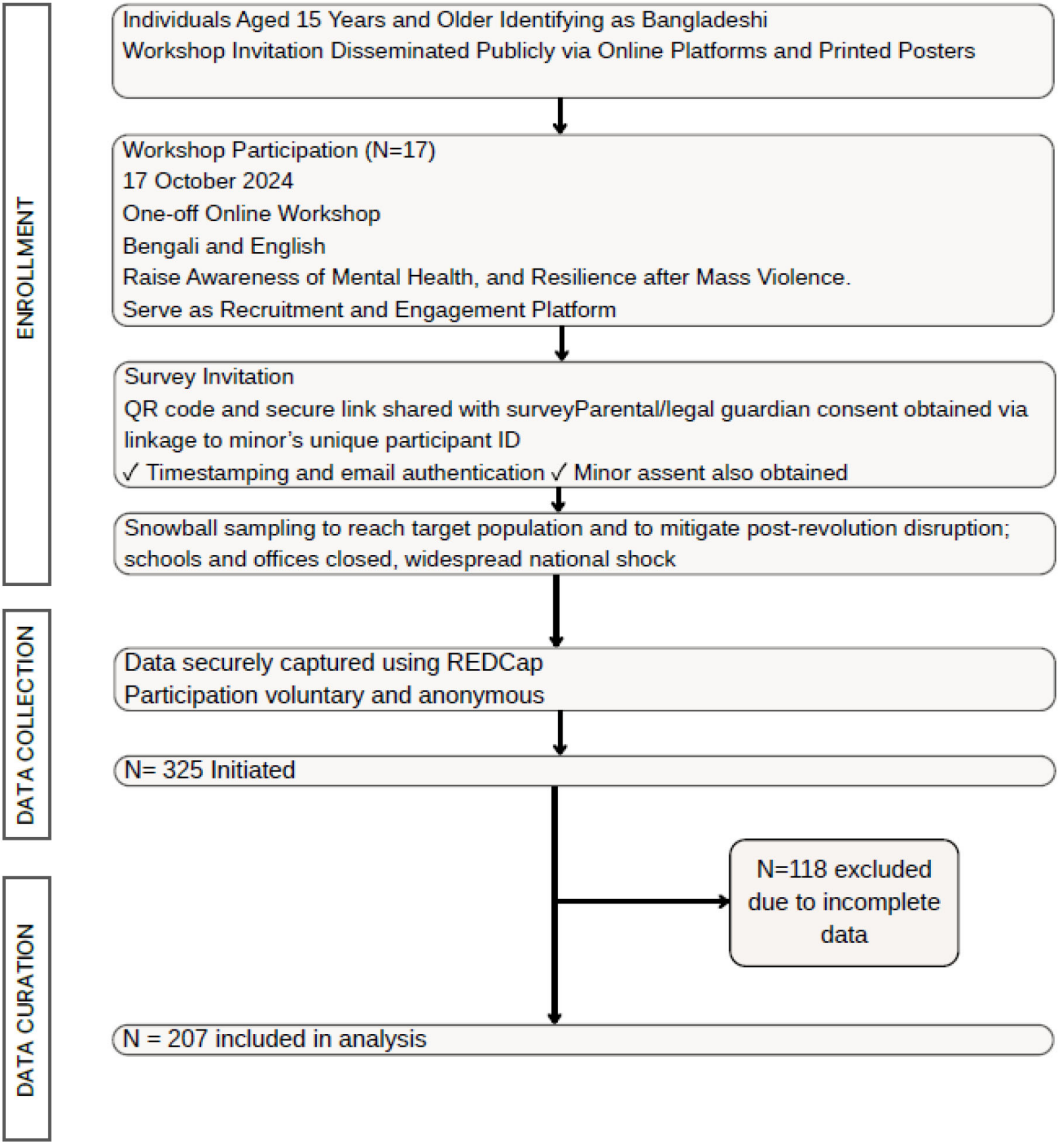
STROBE diagram showing the flow of the recruitment of participants.

Individuals aged fifteen years and above who self-identified as Bangladeshi were invited to participate. The lower age limit of fifteen was selected to inclusively represent the population, acknowledging that adolescents were actively involved in the uprising (1, 4), some even lost their lives (30), while ensuring ethical appropriateness for survey participation. Electronic informed consent was obtained from all participants and parental or guardian consent was required for those under 17 years in line with ethical guidelines. Parental or guardian electronic consent form was linked to the minor’s unique participant identification number. This linkage enabled verification of the consenting adult’s identity and ensured that consent corresponded accurately to the relevant participant. Authentication procedures included timestamping, secure data storage, and email verification. In addition to parental consent, assent was obtained from the minor to affirm their voluntary participation. The recruitment process is shown in the STROBE (STrengthening the Reporting of OBservational studies in Epidemiology) diagram (**Figure 2**).

### Outcome measures

Participants first completed a demographic questionnaire capturing age (date of birth), gender, religion, and division of residence at the time of the events. They then completed the Bangla-translated PTSD Checklist for Diagnostic and Statistical Manual of Mental Disorders, 5th edition (DSM-5) (PCL-5), a 20-item self-report scale assessing symptom severity over the past month. Items were rated on a five-point Likert scale from 0 (“Not at all”) to 4 (“Extremely”), yielding a total score (0-80) and four subscale scores corresponding to DSM-5 Criteria B (re-experiencing), C (avoidance), D (negative alterations in cognition and mood) and E (arousal and reactivity) (31). The Bangla PCL-5 has demonstrated excellent internal consistency (Cronbach’s α = 0.94) and a four-factor structure congruent with DSM-5 criteria. It also showed strong convergent validity with measures of depression and anxiety and robust test-retest reliability over two weeks (intraclass correlation = 0.89) in a representative Bangladeshi adult sample (32). Although DSM-5 guidance recommends a cut-off of 31–33 for probable PTSD (88% sensitivity; 69% specificity), we explored optimal thresholds within our dataset. An open-ended free text box followed the PCL-5, inviting personal reflections. All instruments were piloted to ensure clarity and cultural relevance. The final questionnaire was deployed after ensuring that the questions were easily understandable, as participants had limited opportunity to seek clarification while completing the survey.

### Statistical analysis

Descriptive statistics of sociodemographic characteristics were generated as counts and percentages for categorical variables (age group, gender, religion, and districts) and as means with standard deviations (SD) for continuous variables (total and subscale PCL-5 scores). For unadjusted and adjusted regression analyses, we report mean and mean differences with 95% confidence intervals (CI). Age was categorised into Generation Z (15–24 years), Millennials (25-40), Generation X (41-56) and Boomers (≥ 57), in line with standard cohort definitions.

Between-cluster differences were assessed by Fisher’s exact test for categorical variables (when any cell n < 5) and by the Kruskal-Wallis rank-sum test for continuous outcomes, following assessment of normality via the Shapiro-Wilk test. We examined associations between sociodemographic factors and continuous PCL-5 scores using multiple linear regression models (ANOVA), adjusting for age, gender, religion, and districts (ANCOVA). To evaluate factors associated with probable PTSD (PCL-5 ≥ 31), we fitted log-binomial regression models, preferred over logistic models to avoid overestimation of common outcomes (19) and report prevalence ratios (PR) with 95% CI (33). To ensure the stability of regression estimates, variance inflation factors (VIF) were computed to assess potential multicollinearity among covariates. We calculated population attributable fraction (PAF) and the absolute risk reduction (ARR) in PTSD prevalence under a hypothetical scenario in which high PTSD symptom severity was eliminated. Detailed of the calculation method is in **Supplementary Method A3**. All analyses were conducted in SAS 9.4 (SAS Institute, Cary, NC).

As a complementary analysis, we calculated the min-max normalisation value of the Likert scales of each question (**Supplementary Method A4**). The normalised values for each of the four conceptual domains were used to construct a radar chart to compare the domain-level responses across participants.

### Qualitative analysis

Responses provided in the open-box segment of the survey were set to be analysed using thematic analysis. However, given the limited number and brevity of responses, no formal qualitative analysis was undertaken and responses were reviewed descriptively to complement the quantitative findings.

## Results

**Figure 3:**
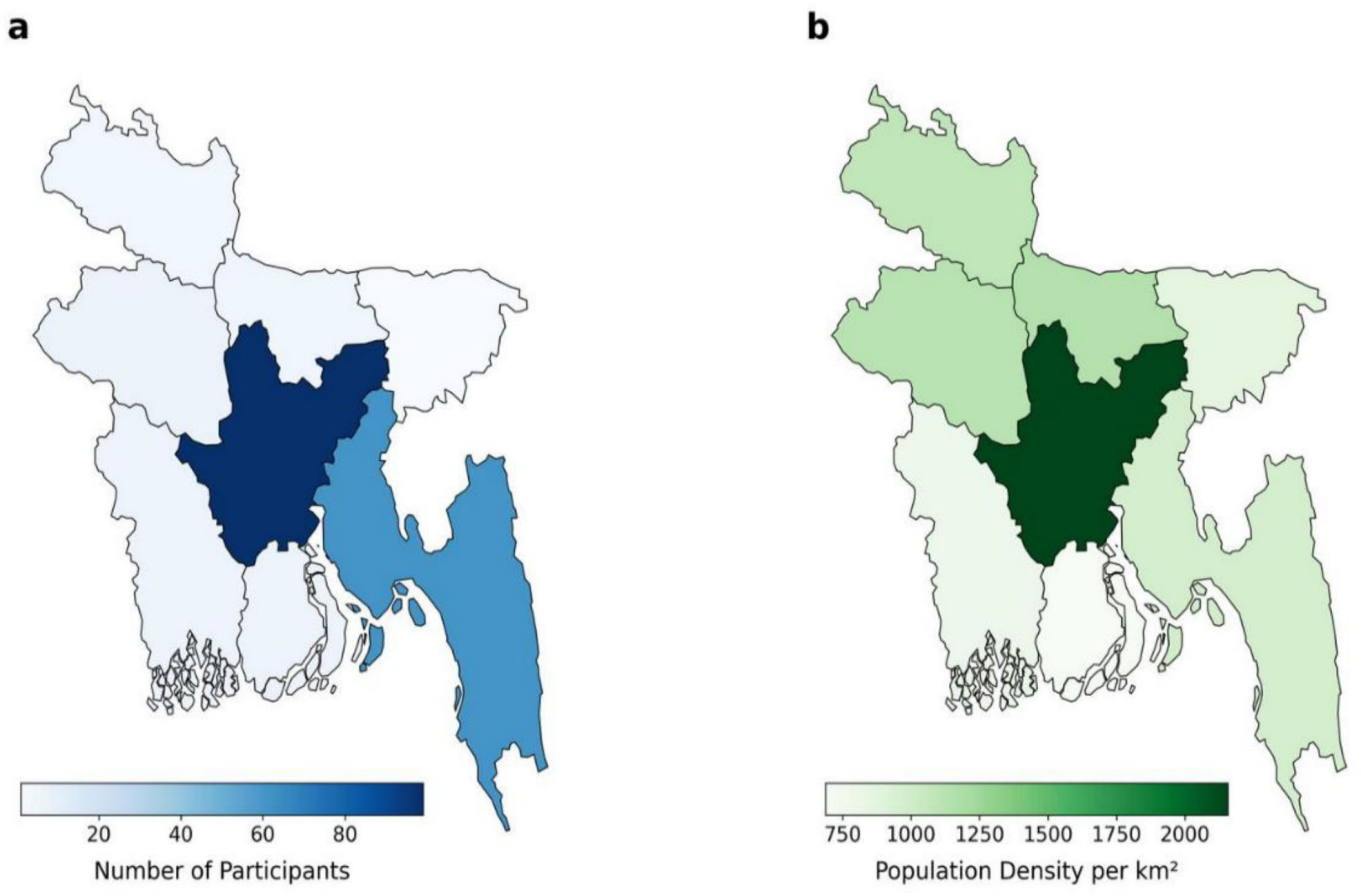
Geographical distribution of the participants of the survey **(A)**, comparing with the population density in Bangladesh **(B)** (census 2022).

This nationwide survey reached 325 participants from all districts in Bangladesh, of which 207 participants completed the survey. Participants who lived in Dhaka and Chattogram during the event were overrepresented in our sample. The **Figure 3** illustrates the geographical distribution of survey respondents across Bangladesh’s eight administrative divisions alongside the 2022 census population density. The left-hand map depicts division‐specific participant counts, overrepresented in Dhaka and Chattogram, while the right‐hand map presents census‐derived population density per square kilometre, highest in Dhaka. We applied raking (**Supplementary Method A5**) to examine the distribution of a given variable shifts after weighting (**Supplementary Table A1**). The very large effect size for geographic divisions (Cohen’s d = 0.98) suggests that the raking procedure effectively corrected the sample’s regional imbalance and likely reflects distortions introduced by post‐revolution internet disruptions during our online data collection.

### Sociodemographic distribution of participants by divisions

**Figure 4:**
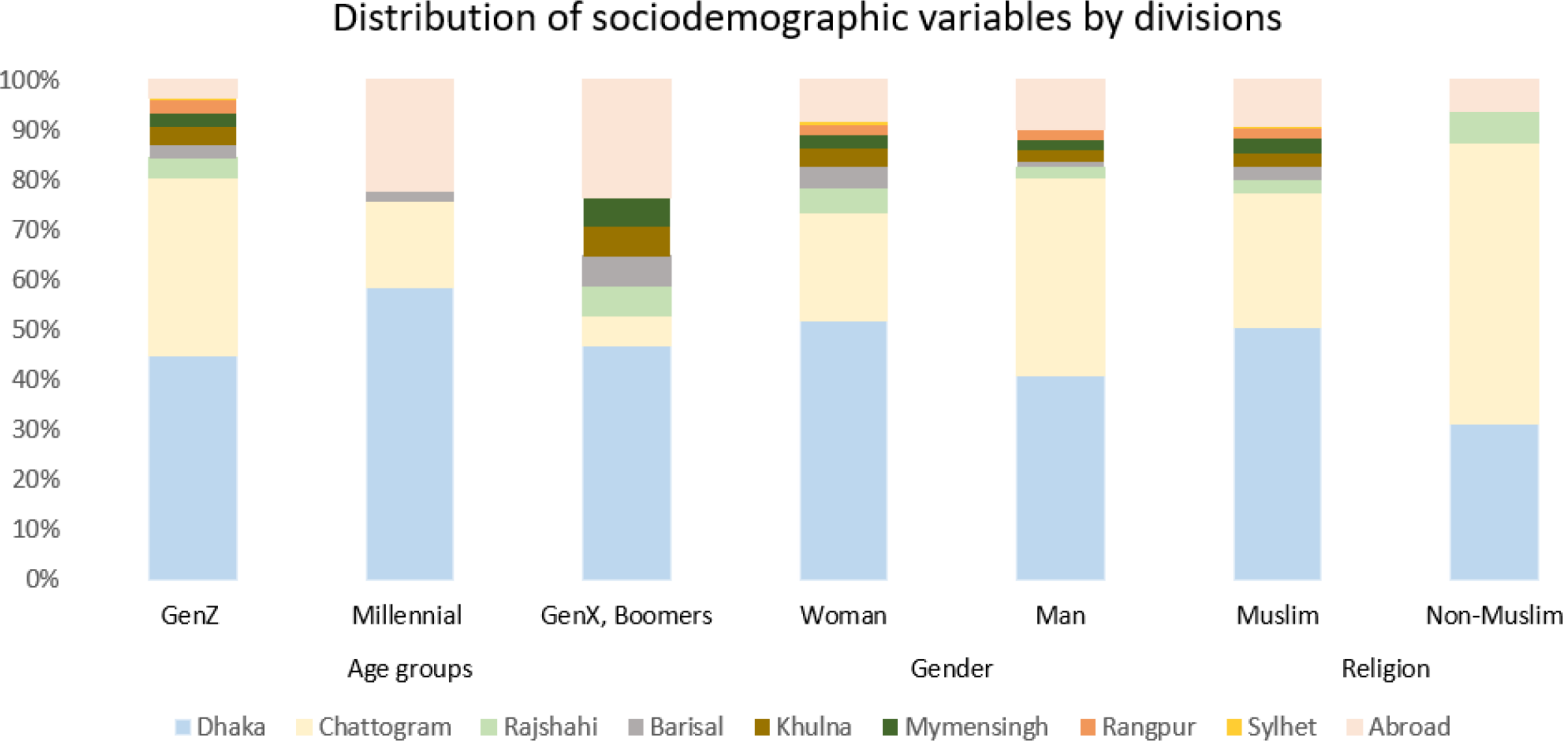
Distribution of sociodemographic variables by divisions of the respondents during the event.

Given that respondents from urban areas were more likely to have direct exposure to and witness the violence, we examined the distribution of demographic variables across divisions to ensure a comprehensive representation, as presented in (**Supplementary Table A4***)*, **Figure 4**.

### Risk factors of PTSD prevalence after revolution

**Figure 5:**
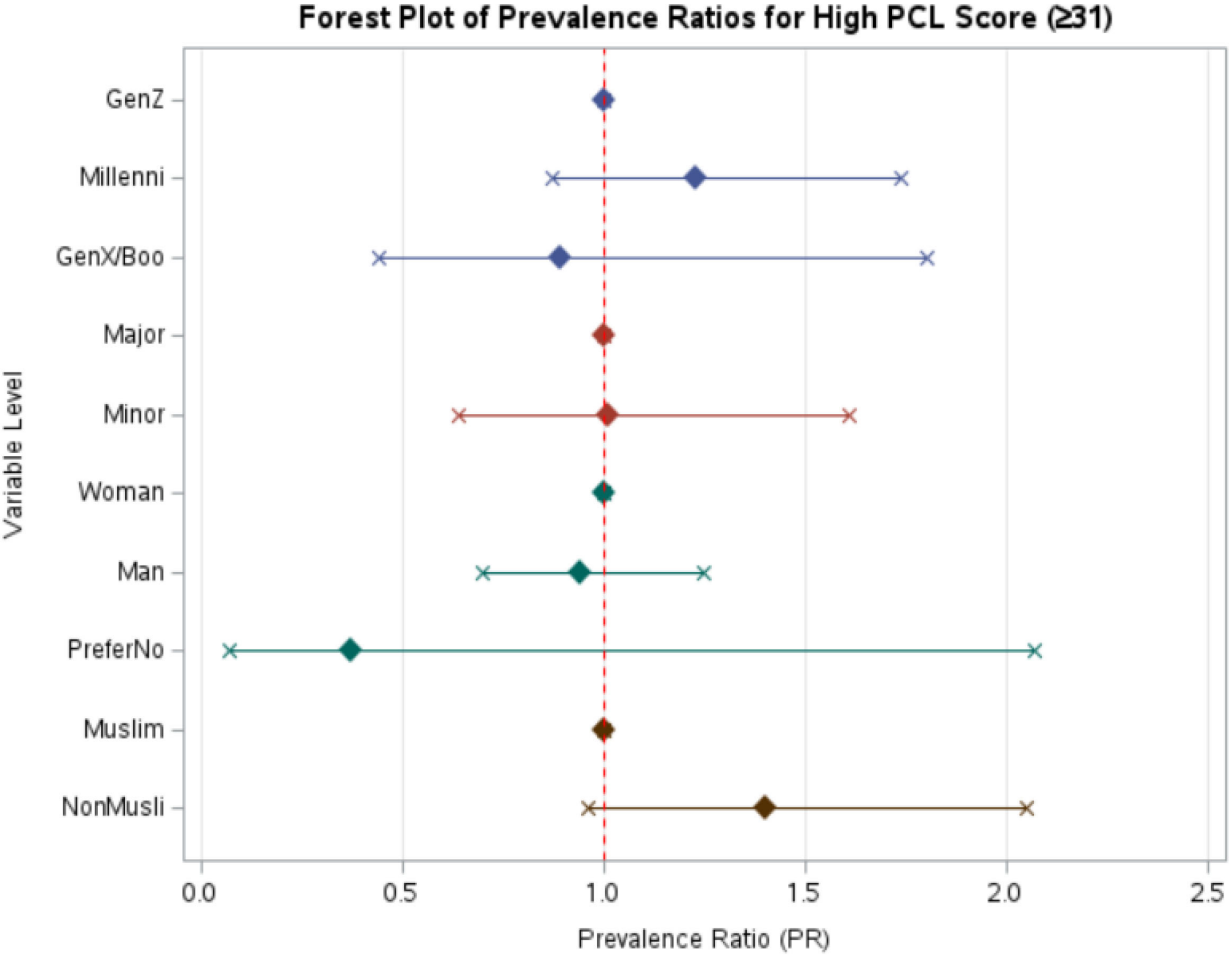
PR with 95% confidence interval showing the association between high PCL score and sociodemographic variables.

The mean age of participants was 27.6 years (± 9.7), with the majority belonging to Generation Z (15–28 years, 72%). Overall, approximately 50% of participants met the cut-off for probable PTSD (PCL ≥ 31). Higher percentages of probable PTSD were observed among women (56.3%), and respondents from Dhaka (43.7) and Chattogram (34%) (**Supplementary Table A2I**). Adjusted analyses suggested modestly higher prevalence among Millennials (PR 1.23, 95% CI 0.87-1.74) and women (**Supplementary Table A3**). Geographic differences were notable, with the proportion of individuals with probable PTSD ranging from 40.0% to 100% across divisions, yielding a highly significant difference (p < 0.0001), whereas divisions showed minimal variation (Minor vs Majors divisions PR 1.01, 0.64-1.61; p = 0.96); **Figure 5**.

### Population attributable fraction and absolute risk reduction for PTSD

**Figure 6:**
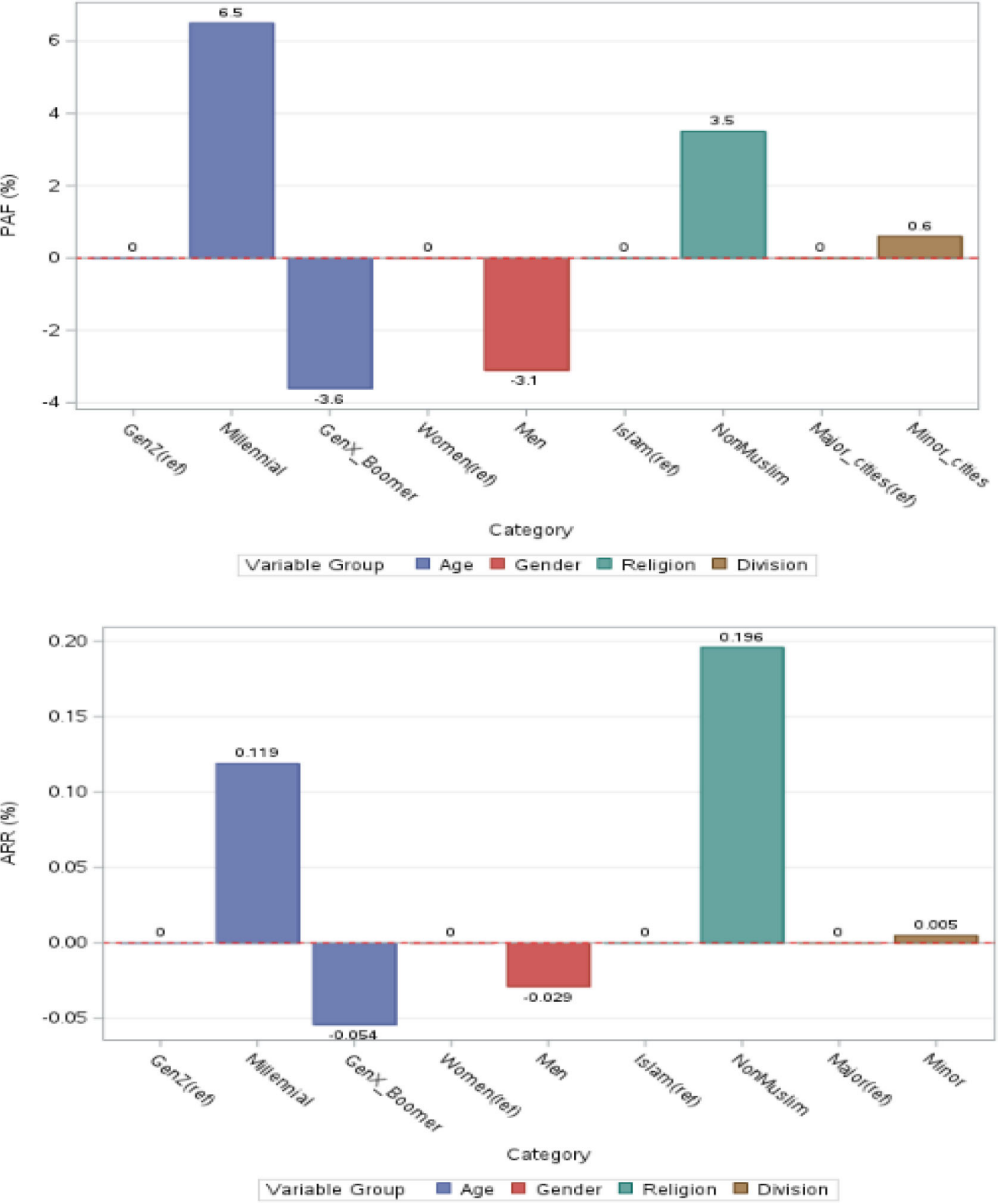
PAF and ARR for PCL-5 adjusting for sociodemographic characteristics.

Results for population attributable fraction are presented in **Supplementary Table A3**; **Figure 6**. Adjusting for sociodemographic characteristics, a small proportion of the outcome was potentially attributable to age, with Millennials showing a modest positive contribution (PAF 6.5%) and Generation X/Boomers a negative contribution (-3.6%). Men had a slightly lower attributable fraction than women (PAF -3.1%), while non-Muslims showed a modest positive contribution (PAF 3.5%). Minor divisions contributed minimally relative to major divisions (PAF 0.6%). If the exposure to the event was hypothetically eliminated the absolute reduction in probable PTSD could be up to 11.9% among Millennials, corresponding to approximately 19.7 million fewer individuals. While the number of GenerationX/Boomers individuals with probable PTSD could increase by 8.9 million. Men could experience a slight increase in probable PTSD, corresponding to 4.7 million additional individuals, whereas non-Muslims could experience a larger absolute reduction of 19.6%, corresponding to 32.4 million fewer individuals. Minor divisions showed minimal impact, with an absolute reduction of 0.5%, corresponding to approximately 0.8 million fewer individuals.

### Mean (SD) scores of PCL-5 clusters across variables

**Figure 7:**
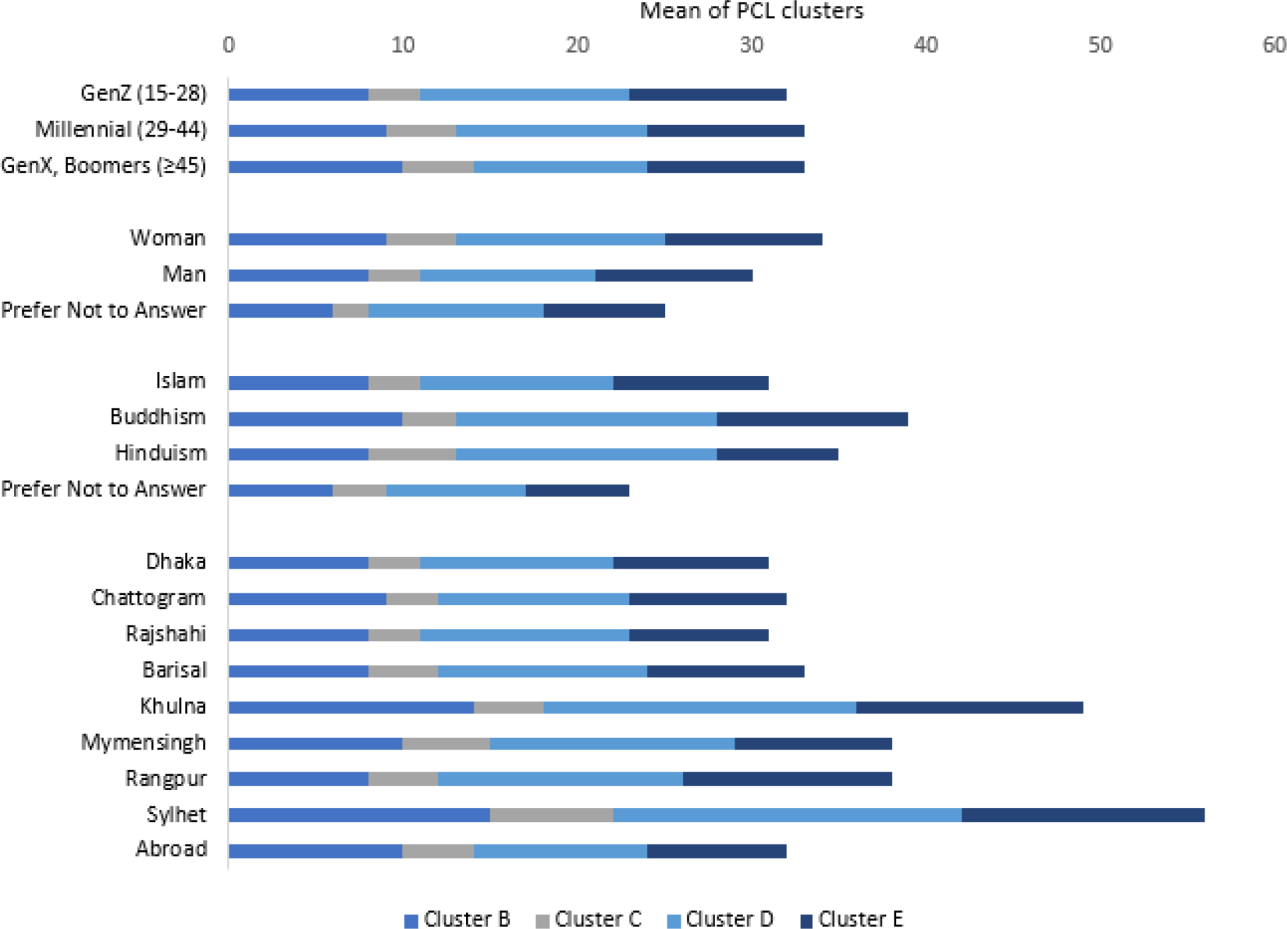
Mean values of PCL clusters by variables.

Individuals aged ≥45 years, women, Buddhists, and respondents from minor division (e.g., Khulna) showed higher scores in Cluster B (re-experiencing). For Cluster C (avoidance), higher scores were observed among those ≥29 years, women (p = 0.01), Hindus, and respondents from minor division (e.g., Mymensingh). Cluster D (negative thoughts/feelings) was elevated among Generation Z, women, non-Muslims. Cluster E (arousal/reactivity) was similar across sociodemographic groups. Overall, women, non-Muslims and Generation X and the Baby Boomer respondents demonstrated higher scores across all symptom clusters **Supplementary Table A5**; **Figure 7**. While Generation Z showed higher scores higher in negative thoughts/feelings (Cluster D).

*Cluster B: Re-experiencing intrusive memories and flashbacks; Cluster C: Avoiding reminders; Cluster D: Negative thoughts and feelings; Cluster E: Alterations in arousal and reactivity.*

### Normalised value of each question within each cluster

**Figure 8:**
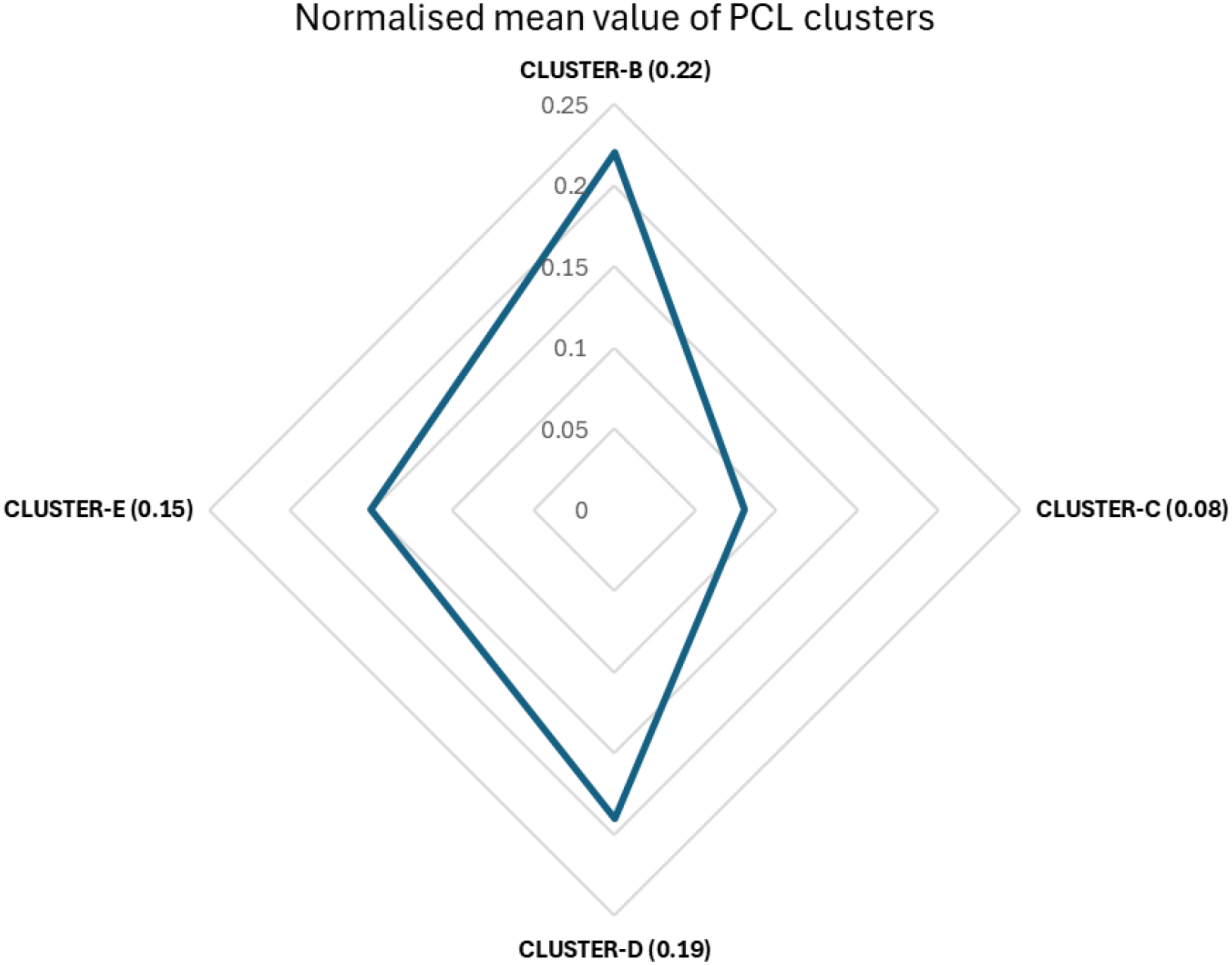
Normalised mean value of Likert scale PCL scores by cluster breakdown.

The normalised values of each question within each cluster supported the mean calculation. The pooled values identified clusters based on higher levels of expressed symptoms, indicating that participants predominantly experienced re-experiencing memories and flashbacks (normalised value: 0.22), followed by heightened negative thoughts and feelings (normalised value: 0.19) (**Supplementary Table A6**; **Figure 8**). The highest scores were observed for feeling upset when reminded of the event (normalised value: 0.31), followed by difficulty concentrating (normalised value: 0.26) and experiencing repeated, disturbing, and unwanted memories of the event.

### ANOVA and ANCOVA in total PCL-5 scores

A series of general linear models (GLM) (**Supplementary Table A7**) were conducted to examine unadjusted and adjusted differences in total PCL-5 scores across sociodemographic groups. In the unadjusted models, mean PCL-5 scores were similar across age groups, with Millennials (M = 33.8, 95% CI 26.9-38.7) scoring slightly higher than Generation Z and Generation X/Boomers. Women reported higher unadjusted scores (M = 34.2, 95% CI 30.6-37.8) compared with men (M = 30.3, 95% CI 26.5- 34.2), though this difference narrowed in adjusted models. Non-Muslims also scored higher than Muslims in both unadjusted and adjusted models. Respondents from minor divisions reported higher unadjusted scores compared with those from major divisions, with a similar pattern after adjustment. Consistent with these findings, in the multivariable ANCOVA, these variables explained only 3% of the variance in total PCL-5 scores (F values all<2.0, R²≤ 0.03) (**Supplementary Table A8**). Overall, the limited proportion of variance accounted for by demographic variables indicates that trauma exposure, rather than demographic characteristics, constitutes the principal determinant of PTSD symptoms.

### Descriptive analyses of qualitative data

The open-ended section of the survey received a range of responses. Among them, 28 participants expressed appreciation for the initiative, while three suggested incorporating more options to assess their level and mode of exposure. Some respondents used this opportunity to share personal experiences, offering valuable insights into the psychological impact of the events that complement the findings shown above. For example, one Generation Z male participant reflected on his experience, stating:

“Basically, the incidents happened in front of my own eyes bother me the most-as if I could have saved the boy, I could have run the other way, or I should have stopped a girl from going forward.”

Another Gen Z male recounted a moment of confrontation:

“I was injured; I was shot by a rubber bullet. I was standing in front of the police, who were holding guns. I thought I would die that day. I was afraid, but I also wanted to be a martyr. There is no negative feeling about that. On the other hand, I found my ambition-now I work hard and enjoy my life very well.”

A Millennial male participant shared how his religious beliefs played a role in his coping mechanism. In his words:

*“I am Muslim, and that’s the reason I don’t feel stress for a long time. When I feel stress, I pray.”* These qualitative responses provide additional depth and personal context to the quantitative results described above. These narratives reveal a spectrum of responses from trauma and survivor’s guilt to resilience, further signalling the salience of direct exposure, emotional processing, and culturally grounded coping strategies in shaping psychological responses.

### Strengths and limitations

A major strength of this study lies in its timing, being conducted within six months of the traumatic event. This proximity enhanced the accuracy of self-reported symptoms by capturing early psychological responses and minimising recall bias. The study also achieved nationwide coverage, with participants from all districts of Bangladesh. Although Dhaka and Chattogram were initially overrepresented, raking adjustments effectively corrected regional imbalances (Cohen’s d = 0.98), enhancing the generalisability of the findings. Furthermore, the final sample size (n = 207) exceeded the calculated requirement (n = 139), strengthening the statistical power and reliability of the results.

The survey was voluntary and accessible to the general population in both Bengali and English, and was complemented by a workshop providing information on the scientific basis of PTSD and the benefits of treatment. Data collection was conducted through a secure, blinded online platform incorporating Google CAPTCHA to prevent invalid submissions, with all PCL-5 items designated as mandatory to minimise missing data. The integration of qualitative and quantitative approaches further enhanced the study’s capacity to capture both individual-level experiences and broader psychosocial dynamics in the post-revolution context.

Nevertheless, this study is not without limitations. The use of snowball sampling, combined with a limited survey administration period, may have introduced selection bias by disproportionately recruiting participants from social networks and demographic groups, particularly younger individuals residing in Dhaka. Such recruitment patterns may have led to the over-representation of certain populations while under-representing others, thereby potentially limiting the generalisability of the findings. Additionally, the relatively small overall sample size, along with limited representation of specific demographic groups such as non-Muslims and older generations, may have reduced statistical power and increased the likelihood of Type II errors. The absence of Christian representatives in this survey constitutes a further limitation, particularly given that Christians make up approximately 0.3% of Bangladesh’s population (34). Despite several attempts to engage potential Christian respondents, these individuals were either not reachable through the recruitment networks or chose not to disclose their religious affiliation, as responding to the question regarding religious belief was voluntary. The online nature of the survey also imposed methodological constraints. Participation required internet access and digital literacy, which may have excluded individuals from rural or socioeconomically disadvantaged backgrounds who have limited access to digital platforms. This limitation may partly explain the relatively high proportion of incomplete responses. One of the key limitations of this self-reported data are subject to response biases, including social desirability and the potential manipulation or exaggeration of responses. Lastly, the absence of funding posed a significant challenge for data collection. Resource constraints limited the ability to organise in-person workshops or community engagement activities that might have facilitated broader and more diverse participation. Similarly, the recruitment of trained field researchers was not feasible due to financial limitations, thereby restricting the scale and reach of the data collection process.

## Discussion

This study identified a PTSD prevalence of 50.3% among the participants, with modestly elevated rates among Millennials, as well as notable geographic and intergenerational variation. This prevalence is substantially higher than those reported in other mass protest contexts (typically range from 4% to 41%), according to a systematic review (22). The comparatively higher prevalence observed in this study may be attributable to the violent nature of the event, together with the predominantly urban and directly exposed composition of the participant sample, both of which are likely to have contributed to an increased psychological burden. The adjusted PR identified that women were more likely to score above the clinical cut-off, with only a marginal difference compared with men (PR = 0.94). These findings are supported by previous literature, which suggests that women are more likely to report symptoms of PTSD following traumatic events due to a combination of biological, psychological, and social factors (22, 35, 36). In contrast, men are more likely to develop stress and anxiety in the longer term due to direct and risky exposure (15). This finding indicates that both genders experienced significant trauma, with men may have experienced comparable levels of trauma despite reporting fewer symptoms. Therefore outreach and intervention efforts should be inclusive without presuming differential coping mechanisms based on gender. A disparity by religious affiliation was observed with non-Muslim participants showed a higher PTSD symptom burden. While not statistically significant after adjustment, this trend may reflect minority stress or sociocultural vulnerability. These findings highlighted the dual role of religious beliefs as both a coping mechanism and a factor in the complex interplay of faith, trauma, and social inequality (37, 38). The revolution was primarily driven by demands for quota reform, contributed to tensions affecting religious minority groups (2). However, given our small sample of non-Muslims, we interpret these findings with caution. Geographical patterns in trauma were pronounced. Respondents from Dhaka and Chattogram had the highest PTSD rates in the country, about two-thirds (77%) screened positive. This suggests a dose-response effect, where closer proximity to the revolution’s most violent episodes reflected in greater psychological impact. Concentrating resources in these “trauma epicentres” could be the most effective way to address the population-level aftermath.

Regarding the comparison of intergenerational levels of stress, Millennials and Generation Z reported higher symptom burden, crossing the clinical threshold for the likely presence of PTSD. This pattern aligns with evidence suggesting that younger populations may be particularly vulnerable to upheaval and violence. Developmentally younger adults may have fewer coping resources for extreme stress, and the 2024 revolution was likely constituted one of the most significant traumatic events experienced by this cohort. Many Boomers and Generation X have lived through multiple national crises (political violence, past movements, even the Liberation War 1971, the 1974 famine, the 1990 mass uprising against military rule, and numerous recurrent natural hazards), which might have fostered a degree of resilience (39–41). In this study, these generations demonstrated comparatively greater resilience in Cluster D, while Generation Z exhibited high levels of agitation and negative thoughts about the future and life. These findings suggest that while older generations may demonstrate greater resilience, younger generation faces unique mental health challenges, including increased agitation and negative thoughts about the future, supported by previous studies (42, 43).

Another important aspect of the 2024 revolution is its collective nature, which influenced how people experience trauma. Many participants described their struggles in terms of a shared national cause rather than purely personal loss. The protests created a powerful sense of unity and purpose among the students and citizens, contributing to the formation of a shared collective identity. This solidarity may have provided a psychological buffer for some individuals. The literature on collective trauma suggests that when individuals perceive a shared collective experience it can mitigate feelings of helplessness and isolation. Some respondents in our study expressed pride or meaning in their sacrifice, which might have facilitated psychological coping with distress. However, collective euphoria may have complex or paradoxical effects. If the movement’s goals are perceived as unfulfilled or if some people feel alienated in the aftermath, that can lead to frustration or a sense of betrayal, potentially exacerbating trauma. Our qualitative notes captured this dichotomy. A few injured protesters reported they found personal growth or newfound purpose from the experience, whereas others voiced despair or survivor’s guilt (e.g., “Why did I survive when my friend did not?”). These mixed emotional responses illustrate that a revolution’s psychological legacy is complex. The “Revolution’s aftermath” is thus not just about individual PTSD rates, but also about how a society makes meaning of a period of significant collective upheaval. Psychologically, revolutions often function as catalysts for collective identity formation. As theorised in social psychology, mass movements generate a sense of unity in which individual consciousness dissolves into the larger group identity. Revolutions do not merely reshape political landscapes; they also produce substantial psychological effects on the emotional and psychological state of individuals.

We recommend further research to monitor and support the long-term recovery. A cross-sectional survey cannot fully capture how mental health trajectories evolve after a mass trauma. It will be valuable to conduct a longitudinal study following this cohort (and possibly a broader population sample) over subsequent years. Such a study could determine whether the initially high PTSD rates persist, improve, or deteriorate (e.g., through delayed-onset cases). It would also help identify predictors of resilience versus chronic distress – for instance, do people who receive counselling recover faster? Does community cohesion or economic rehabilitation in certain areas correlate with improved mental health outcomes? Additionally, future research could explore biological markers or qualitative narratives to enrich our understanding of the revolution’s psychological impact. Continued surveillance and inquiry will guide more effective, evidence-based interventions.

## Data Availability

The raw data supporting the conclusions of this article will be made available by the authors, without undue reservation.
