# Supplementary material for: Revolution’s aftermath: population based cross-sectional study to understand the intergeneration mental health and wellbeing following the 2024 student-led uprising
Source: Front Psychiatry. 2026 May 12;17:1824190. doi: 10.3389/fpsyt.2026.1824190 (PMC13201506; doi:10.3389/fpsyt.2026.1824190)
Supplement: Supplementary file 1 [file Supplementaryfile1.docx]

**Revolution's Aftermath: Population Based Cross-Sectional Study to Understand the Intergeneration Mental Health and Wellbeing Following the 2024 Student-Led Uprising.**

Table of Contents

[SUPPLEMENTARY METHODS 3](#_Toc211549437)

[Supplementary Method 1. Steps in recruiting participants 3](#_Toc211549438)

[Supplementary Method 2. Sample size calculation 4](#_Toc211549439)

[Supplementary Method 3. Calculation of population attributable fraction 4](#_Toc211549440)

[Supplementary Method 4. Calculation of normalised value of Likert scale 5](#_Toc211549441)

[Supplementary Method 5. Calculation of raking 5](#_Toc211549442)

[SUPPLEMENTARY RESULTS 7](#_Toc211549443)

[Table 1: Sociodemographic composition of the study participants in the weighted sample compared to 2022 Bangladesh census 7](#_Toc211549444)

[Table 2 Sociodemographic characteristics and PCL-5 score cut-off number with percentages of participants. 8](#_Toc211549445)

[Table 3 PR with 95% confidence interval, PAF and ARR showing the association between high PCL score and sociodemographic variables. 9](#_Toc211549446)

[Table 4: Distribution of age, gender and religious belief by the location of the respondents during the event. 10](#_Toc211549447)

[Table 5: Mean (SD) values of PCL clusters by variables 11](#_Toc211549448)

[Table 6: Normalised values of PCL-5 scores 12](#_Toc211549449)

[Table 7: Adjusted (ANCOVA) and crude (ANOVA) regression models show Mean and mean differences value of the total PCL scores by variables. 13](#_Toc211549450)

[Table 8: Univariate and multivariable regression results for predictors of PCL-5 total score LSMean 14](#_Toc211549451)

[REFERENCES 15](#_Toc211549452)

# SUPPLEMENTARY METHODS

## Supplementary Method 1. Steps in recruiting participants

The workshop was conducted on 17 October 2024, approximately two months after the revolution. This timing was selected to allow for initial psychosocial stabilisation and reflection, enabling participants to articulate early mental health responses and resilience strategies in a post-crisis context. The session explained and sensitised the importance of mental health outcomes, trauma, and positive resilience after violent events. The workshop was open to the general population in Bangladesh, with invitations disseminated via online platforms and printed materials. It was delivered in both Bengali and English to accommodate participants in Bangladesh and Bangladeshi expatriates affected by media exposure. The workshop served not only as a recruitment platform but also as a qualitative complement to the survey. It provided contextual insights into community perceptions of trauma and resilience, which informed the interpretation of survey responses and enhanced the ecological validity of the findings. Following the workshop, attendees were invited to participate in an online survey via a secure link and QR code (**Appendix Fig 1**). The survey remained open for one month on social media; with snowball sampling employed to reach a dispersed population. We used online data collection to reach wider population with easy access. As majority of the affected population has online access, we assume a minimal selection bias due to data collection method. Overall, these recruitment strategies were used to reach out to the target population following the socio-political disruption what significantly compromised the feasibility of conventional sampling methods.


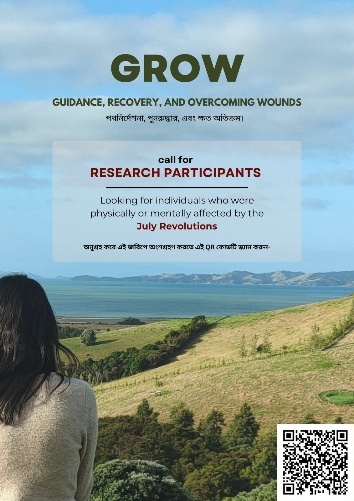


Appendix Fig 1: Poster for survey invitation with online QR code and link.

## Supplementary Method 2. Sample size calculation

Our sample size was calculated based on the Leslie and Kish formula for estimating a proportion in a cross-sectional study [1, 2]: n=$\frac{Z^{2}p\left( 1-p \right)}{d^{2}}$, where n denotes the required sample size, Z is the statistic corresponding to the level of confidence, p is the expected prevalence, and d is the precision (absolute error). A type I error of 0.05 was applied, corresponding to a 95% confidence level (1.96), with the hypothesised prevalence (p) set at 10% [3, 4] in the general population aged ≥15 years in Bangladesh [5]. The precision (d) was set at 5%. Based on these parameters, the calculated minimum sample size was 139 participants.

## Supplementary Method 3. Calculation of population attributable fraction

We used our survey data to estimate the contribution of selected sociodemographic exposures to the prevalence of high psychological distress, defined as a PCL-5 score ≥31. Exposure categories (e.g. age group, gender, religious affiliation, and location) were defined according to standard classifications, and population-level proportions for each category were derived from the 2022 Bangladesh national census [6]. Adjusted prevalence ratios (PRs) for high PCL-5 scores were obtained from multiple logistic regression models, controlling for relevant sociodemographic covariates. The Population Attributable Fraction (PAF) was calculated for each exposure to estimate the proportion of high PTSD in the population attributable to that exposure.

Absolute Risk Reduction (ARR) was calculated as the difference in observed prevalence of high psychological distress between each exposure category and the reference group. To simulate a counterfactual scenario in which the impact of a given exposure was removed, the exposure variable was recoded to its reference level for all participants. Simulated probabilities of high psychological distress were then generated using the same regression model. The absolute reduction in prevalence was estimated by averaging the difference between observed and simulated probabilities across all participants. The PAF was subsequently derived as the average proportion of reduction relative to the observed probability.

## Supplementary Method 4. Calculation of normalised value of Likert scale

To ensure comparability across items measured on Likert scales, we applied min–max normalisation to the mean scores of each question. This technique transforms raw values into a standardised range between 0 and 1, preserving the relative distribution while eliminating scale-related distortions [7]. Specifically, the mean value (µ) of each item was rescaled using the min–max formula, which adjusts each score based on the minimum and maximum observed values within the item’s response range. This approach is particularly suitable for survey data, where Likert-type items may vary in interpretability and scale anchoring across domains [8]. Normalisation facilitates visual comparison and aggregation by aligning all variables to a common scale, thereby reducing bias introduced by differing measurement units or response distributions. Data normalisation is a critical preprocessing step that enhances interpretability and analytical consistency, especially when integrating multiple indicators into composite visualisations [9].

## Supplementary Method 5. Calculation of raking

To ensure that the survey sample reflected the demographic composition of the national population, we applied raking (iterative proportional fitting) to align the marginal distributions of the sample with official Bangladesh Census benchmarks. Raking is widely used in survey methodology to reduce bias due to over- or under-representation of specific demographic groups. The procedure involved calculating census population proportions and converting them into absolute counts, if not available. The survey proportions were then compared with these census margins, and respondent weights were iteratively adjusted until the weighted sample matched the census distributions within a pre-specified tolerance. Weighted sample sizes were obtained by multiplying census proportions by the total sample (N=207), and Cohen’s w effect size was calculated to quantify the discrepancy between the unweighted survey and census distributions. Responses recorded as missing or prefer not to answer were excluded from the raking procedure but retained in descriptive reporting.

# **SUPPLEMENTARY RESULTS**

# Table 1: Sociodemographic composition of the study participants in the weighted sample compared to 2022 Bangladesh census

| Sociodemographic Variables | Bangladesh 2022 Census^a^ % | Weighted sample n (%) | Unweighted sample n (%) | Effect size^b^ |
| --- | --- | --- | --- | --- |
| Age Group |  |  |  |  |
| *GenZ (15-28)* | 61,865,000 (37.5) | 78 (37.5) | 149 (72.0) | 0.73 |
| *Millennial (29-44)* | 50,093,000 (30.3) | 63 (30.3) | 41 (19.8) |  |
| *GenX, Boomers (≥45)* | 53,200,000 (32.2) | 67 (32.2) | 17 (8.2) |  |
| Gender |  |  |  |  |
| *Woman* | 83,347,206 (50.5) | 104 (50.2) | 110 (53.1) | 0.075 |
| *Man* | 81,712,824 (49.5) | 103 (49.8) | 93 (44.9) |  |
| Religious Belief |  |  |  |  |
| *Islam* | 150,433,559 (91.0) | 179 (91.0) | 186 (94.4) | 0.75 |
| *Buddhism* | 1,007,467 (0.6) | 01 (0.6) | 12 (6.1) |  |
| *Hinduism* | 13,130,117 (7.9) | 16 (7.9) | 02 (1.0) |  |
| *Christianity* | 495,476 (0.3) | 01 (0.3) | 0 |  |
| *Others* | ~190,000 (0.1) | 0 (0.1) | 0 |  |
| *Prefer not to say* | - | - | 02 (0.1) |  |
| Divisions of the respondents during the event |  |  |  |  |
| *Dhaka* | 23,447,216 (20.7) | 39 (20.7) | 99 (47.8) | 0.98 |
| *Chattogram* | 22,011,712 (19.5) | 37 (19.5) | 61 (29.5) |  |
| *Rajshahi* | 15,506,337 (13.7) | 26 (13.7) | 07 (3.4) |  |
| *Barisal* | 6,809,844 (6.0) | 11 (6.0) | 06 (2.9) |  |
| *Khulna* | 13,097,250 (11.6) | 22 (11.6) | 06 (2.9) |  |
| *Mymensingh* | 9,489,451 (8.4) | 16 (8.4) | 05 (2.4) |  |
| *Rangpur* | 13,738,138 (12.1) | 23 (12.1) | 04 (1.9) |  |
| *Sylhet* | 8,963,639 (7.9) | 15 (7.9) | 01 (0.5) |  |

^a^ Source: 2022 Census Bangladesh; ^b^ Cohen’s *w* effect size

# Table 2 Sociodemographic characteristics and PCL-5 score cut-off number with percentages of participants.

| Variables | N (Col %) | Total PCL< 30, N (col %) | Total PCL ≥ 31, N (col%) | |
| --- | --- | --- | --- | --- |
| Age Group | | | | |
| *GenZ (15-28)* | 149 (72.0) | 74 (71.2) | 75 (72.8) | |
| *Millennial (29-44)* | 41 (19.8) | 20 (19.2) | 21 (20.4) | |
| *GenX, Boomers (≥45)* | 17 (8.2) | 10 (9.6) | 07 (6.8) | |
| *Missing* | 0 | - | - | |
|  | *p-value***^a^** *<0.0001** | *p-value^b^ = 0.02** | | |
| Gender | | | | |
| *Woman* | 110 (53.1) | 52 (50.0) | 58 (56.3) | |
| *Man* | 93 (44.9) | 49 (47.1) | 44 (42.7) | |
| *Prefer Not to Answer* | 04 (1.9) | 03 (2.9) | 01 (1.0) | |
| *Missing* | 0 | - | - | |
|  | *p-value***^a^** *<0.0001** | *p-value^b^ = 0.02** | | |
| Religious Belief | | | | |
| *Islam* | 186 (92.1) | 98 (94.2) | 88 (89.8) | |
| *Buddhism* | 12 (5.9) | 03 (2.9) | 09 (9.2) | |
| *Hinduism* | 02 (1.0) | 01 (1.0) | 01 (1.0) | |
| *Prefer Not to Answer* | 02 (1.0) | 02 (1.9) | 0 | |
| *Missing* | 05 | - | - | |
|  | *p-value***^a^** *<0.0001** | *p-value^b^ = 0.006** | | |
| Districts of the respondents during the event | | | |  |
| *Dhaka* | 99 (47.8) | 54 (51.9) | 45 (43.7) | |
| *Chattogram* | 61 (29.5) | 26 (25.0) | 35 (34.0) | |
| *Rajshahi* | 07 (3.4) | 04 (3.9) | 03 (2.9) | |
| *Barisal* | 06 (2.9) | 03 (2.9) | 03 (2.9) | |
| *Khulna* | 06 (2.9) | 02 (1.9) | 04 (3.9) | |
| *Mymensingh* | 05 (2.4) | 03 (2.9) | 02 (1.9) | |
| *Rangpur* | 04 (1.9) | 02 (1.9) | 02 (1.9) | |
| *Sylhet* | 01 (0.5) | 0 | 01 (1.0) | |
| *Abroad* | 18 (8.7) | 10 (9.6) | 08 (7.8) | |
| *Missing* | 0 | - | - | |
|  | *p-value***^a^** *<0.0001** | *p-value^b^ <0.0001** | | |

^a^ Fisher's Exact Test showing group differences between participants, *^b^* P-value for differences in total score between all levels of variable

# Table 3 PR with 95% confidence interval, PAF and ARR showing the association between high PCL score and sociodemographic variables.

| **Variables with levels** | **PR with 95% CI of high PCL score**^a^ | **Exposure Prevalence**^b^ **(%)** | **Population attributable fraction (PAF, %)** | **Absolute Risk Reduction (ARR, %)** |
| --- | --- | --- | --- | --- |
| **Age groups (years)** |  |  |  |  |
| GenZ | 1.00 | 37.5 | 0 | 0 |
| Millennial | 1.23 (0.87, 1.74) | 30.3 | 6.5 | 0.119 |
| GenX, Boomers | 0.89 (0.44, 1.80) | 32.2 | -3.6 | -0.054 |
|  | *p-value = 0.50* |  |  |  |
| **Gender** |  |  |  |  |
| Woman | 1.00 | 50.5 | 0 | 0 |
| Man | 0.94 (0.70, 1.25) | 49.5 | -3.1 | -0.0287 |
| Prefer Not to say | 0.37 (0.07, 2.07) | - | - |  |
|  | *p-value = 0.28* |  |  |  |
| **Religious belief** |  |  |  |  |
| Muslim | 1.00 | 91.0% | 0 | 0 |
| Non-Muslim | 1.40 (0.96, 2.05) | 9.0% | 3.5 | 0.196 |
|  | *p-value = 0.15* |  |  |  |
| **Divisions** |  |  |  |  |
| Major | 1.00 | 40.2 | 0 | 0 |
| Minor | 1.01 (0.64, 1.61) | 59.8 | 0.6 | 0.005 |
|  | *p-value = 0.96* |  |  |  |

^a^ Adjusted for all variables in the table. ^b^ Source: 2022 Census Bangladesh

# Table 4: Distribution of age, gender and religious belief by the location of the respondents during the event.

| Variables with levels | Divisions during the event N (Row %) | | | | | | | | | | | | | | | | | |
| --- | --- | --- | --- | --- | --- | --- | --- | --- | --- | --- | --- | --- | --- | --- | --- | --- | --- | --- |
|  | **Dhaka** | | | **Chattogram** | | **Rajshahi** | | **Barisal** | **Khulna** | | **Mymensingh** | | **Rangpur** | | **Sylhet** | | **Abroad** | |
| Age groups (years) |  | | | | | | | | | | | | | | | | | |
| GenZ (15-28) | | | 67 (45.0) | 53 (35.6) | | 06 (4.0) | | 04 (2.7) | | | 05 (3.4) | | 04 (2.7) | | 04 (2.7) | 01 (0.7) | 05 (3.4) | |
| Millennial (29-44) | | | 24 (58.6) | 07 (17.1) | | 0 | | 01 (2.4) | | | 0 | | 0 | | 0 | 0 | 09 (22.0) | |
| GenX, Boomers (≥45) | | | 08 (47.1) | 01 (5.9) | | 01 (5.9) | | 01 (5.9) | | | 01 (5.9) | | 01 (5.9) | | 0 | 0 | 04 (23.5) | |
| *p <0.0001** | | |  |  | |  | |  | | |  | |  | |  |  |  | |
| Gender |  | | | | | | | | | | | | | | | | | |
| Woman | 57 (51.8) | | | 24 (21.8) | | 05 (4.6) | | 05 (4.6) | 04 (3.6) | | 03 (2.7) | | 02 (1.8) | | 01 (0.9) | | 09 (8.2) | |
| Man | 38 (40.9) | | | 37 (39.8) | | 02 (2.2) | | 01 (1.1) | 02 (2.2) | | 02 (2.2) | | 02 (2.2) | | 0 | | 09 (9.7) | |
| Prefer Not to say | 04 (100.0) | | | 0 | | 0 | | 0 | 0 | | 0 | | 0 | | 0 | | 0 | |
| *p <0.0001** |  | | |  | |  | |  |  | |  | |  | |  | |  | |
| Religious belief |  | | |  | |  | |  |  | |  | |  | |  | |  | |
| Muslim | 94 (50.5) | | | 50 (26.9) | | 05 (2.7) | | 05 (2.7) | 05 (2.7) | | 05 (2.7) | | 04 (2.2) | | 01 (0.5) | | 17 (9.1) | |
| Non-Muslim | 05 (31.3) | | | 09 (56.3) | | 01 (6.3) | | 0 | 0 | | 0 | | 0 | | 0 | | 01 (6.3) | |
| *p=0.0005** |  | | |  | |  | |  |  | |  | |  | |  | |  | |

*Statistically significant (P values from Monte Carlo’s Fisher Exact tests to compare the distributions of variables)

# Table 5: Mean (SD) values of PCL clusters by variables

| Variables | Mean (SD) of Cluster B | Mean (SD) of Cluster C | Mean (SD) of Cluster D | Mean (SD) of Cluster E |
| --- | --- | --- | --- | --- |
| Age Group | | | | |
| GenZ (15-28) | 8 (5) | 3 (2) | 12 (8) | 9 (6) |
| Millennial (29-44) | 9 (6) | 4 (3) | 11 (8) | 9 (7) |
| GenX, Boomers (≥45) | 10 (6) | 4 (3) | 10 (7) | 9 (7) |
| *p-value^#^* | *0.60* | *0.45* | *0.61* | *0.91* |
| Gender | | | | |
| Woman | 9(6) | 4 (2) | 12 (8) | 9 (6) |
| Man | 8 (5) | 3 (2) | 10 (8) | 9 (6) |
| Prefer Not to Answer | 6 (3) | 2 (2) | 10 (4) | 7 (4) |
| *p-value^#^* | *0.52* | *0.01** | *0.14* | *0.68* |
| Religious Belief | | | | |
| Islam | 8 (5) | 3 (2) | 11 (8) | 9 (6) |
| Buddhism | 10 (5) | 3 (2) | 15 (5) | 11 (5) |
| Hinduism | 8 (9) | 5 (2) | 15 (14) | 7 (7) |
| Prefer Not to Answer | 6 (1) | 3 (1) | 8 (6) | 6 (6) |
| *p-value^#^* | *0.66* | *0.87* | *0.34* | *0.58* |
| Location of the respondents | | | | |
| Dhaka | 8 (5) | 3 (3) | 11 (8) | 9 (6) |
| Chattogram | 9 (5) | 3 (2) | 11 (7) | 9 (5) |
| Rajshahi | 8 (5) | 3 (1) | 12 (9) | 8 (4) |
| Barisal | 8 (8) | 4 (3) | 12 (12) | 9 (10) |
| Khulna | 14 (6) | 4 (3) | 18 (8) | 13 (6) |
| Mymensingh | 10 (6) | 5 (2) | 14 (6) | 9 (5) |
| Rangpur | 8 (4) | 4 (3) | 14 (11) | 12 (6) |
| Sylhet | 15 (-) | 7 (-) | 20 (-) | 14 (-) |
| Abroad | 10 (6) | 4 (2) | 10 (7) | 8 (7) |
| *p-value^#^* | *0.10* | *0.62* | *0.49* | *0.76* |

^#^ Fisher's Exact Test showing group differences between participants, SD = standard deviation *Statistically significant.

# Table 6: Normalised values of PCL-5 scores

| **PCL Clusters with questions** | **Mean Value** | **Normalised Value** |
| --- | --- | --- |
| **CLUSTER B: Re-experiencing intrusive memories and flashbacks** | | |
| PCL_01 Repeated, disturbing, and unwanted memories of the stressful experience? | 2.03 | 0.26 |
| PCL_02 Repeated, disturbing dreams of the stressful experience? | 1.32 | 0.08 |
| PCL_03 Suddenly feeling or acting as if the stressful experience were actually happening again | 1.6 | 0.15 |
| PCL_04 Feeling very upset when something reminded you of the stressful experience? | 2.23 | 0.31 |
| PCL_05 Having strong physical reactions when something reminded | 1.41 | 0.1 |
| **CLUSTER C: Avoiding reminders** | | |
| PCL_06 Avoiding memories, thoughts, or feelings related to the stressful experience | 1.76 | 0.19 |
| PCL_07 Avoiding external reminders of the stressful experience | 1.58 | 0.14 |
| **CLUSTER D: Negative Thoughts and Feelings** | | |
| PCL_08 Trouble remembering important parts of the stressful experience? | 1.29 | 0.07 |
| PCL_09 Having strong negative beliefs about yourself, other people, or the world | 1.74 | 0.18 |
| PCL_10 Blaming yourself or someone else | 1.63 | 0.16 |
| PCL_11 Having strong negative feelings such as fear, horror, anger, guilt, or shame | 1.71 | 0.18 |
| PCL_12 Loss of interest in activities that you used to enjoy? | 1.67 | 0.17 |
| PCL_13 Feeling distant or cut off from other people | 1.77 | 0.19 |
| PCL_14 Trouble experiencing positive feelings | 1.5 | 0.12 |
| **CLUSTER E: Alterations in arousal and reactivity** | | |
| PCL_15 Irritable behavior, angry outbursts, or acting aggressively | 1.59 | 0.15 |
| PCL_16 Taking too many risks or doing things that could cause you harm | 1.13 | 0.03 |
| PCL_17 Being 'super alert' or watchful or on guard | 1.52 | 0.13 |
| PCL_18 Feeling jumpy or easily startled | 1.18 | 0.05 |
| PCL_19 Having difficulty concentrating | 2.05 | 0.26 |
| PCL_20 Trouble falling or staying asleep | 1.62 | 0.16 |

# Table 7: Adjusted (ANCOVA) and crude (ANOVA) regression models show Mean and mean differences value of the total PCL scores by variables.

| **Variables** | | **Unadjusted total score of PCL-5** | | | | **Adjusted^#^ total score of PCL-5** | | | |
| --- | --- | --- | --- | --- | --- | --- | --- | --- | --- |
|  | | Mean (95% CI) | | Mean Diff (95% CI) | | Mean (95% CI) | | Mean Diff (95% CI) | |
| **Age Group** | | | | | | | | | |
| GenZ (15-28) | | 32.2 (29.1, 35.3) | | Ref | | 32.4 (23.9, 41.0) | | Ref | |
| Millennia**l** (29-44) | | 33.8 (26.9, 38.7) | | 0.61 (-6.0, 7.2); p=0.86 | | 36.1 (26.8, 45.5) | | 3.7 (-3.8, 11.2); p=0.33 | |
| GenX, Boomers (≥45) | | 31.8 (22.6, 40.9) | | -0.41 (-9.9, 9.1); p=0.93 | | 31.4 (17.9, 44.8) | | -1.1 (-12.2, 10.0); p=0.85 | |
| **Gender** | | | | | | | | | |
| Woman | | 34.2 (30.6, 37.8) | | Ref | | 37.7 (30.8, 44.5) | | Ref | |
| Man | | 30.3 (26.5, 34.2) | | -3.86 (-9.1, 1.3); p=0.15 | | 35.9 (28.1, 43.7) | | -1.8 (-7.4, 3.8); p=0.53 | |
| Prefer Not to Answer | | 24.0 (5.3, 42.7) | | -10.19 (-29, 8.6); p=0.29 | | 26.3 (6.5, 46.2) | | -11.4 (-30.5, 7.8); p=0.24 | |
| **Religious Belief** | | | | | | | | | |
| Muslim | | 31.6 (26.6, 45.3) | | Ref | | 30.4 (22.6, 38.2) | | Ref | |
| Non-Muslims | | 35.9 (26.6, 45.3) | | 4.32 (-5.3, 14.0); p=0.38 | | 36.2 (24.3, 48.1) | | 5.8 (-4.3, 15.8); p=0.26 | |
| **Location of the respondent** | | | | | | | | | |
| Major Divisions | | 31.2 (28.3, 34.2) | | Ref | | 30.5 (22.4, 38.7) | | Ref | |
| Minor Divisions | | 37.9 (31.0, 44.8) | | 6.7 (-0.7, 14.1); p=0.07 | | 36.1 (25.3, 46.9) | | 5.6 (-2.3, 13.5); p=0.16 | |
| Abroad | | 32.4 (23.5, 41.2) | | Not included | | Not included | | Not included | |

# Adjusted for all variables in the table.

# Table 8: Univariate and multivariable regression results for predictors of PCL-5 total score LSMean

|  | **Univariate ANOVA** | | | | **Multivariable ANCOVA** | | | |
| --- | --- | --- | --- | --- | --- | --- | --- | --- |
| **Variables** | **df** | **F** | **p** | **R²** | **df** | **F** | **p** | **R²** |
| **Age group** | 2 | 0.02 | 0.978 | 0.0002 | 2 | 0.49 | 0.612 | 0.03 |
| **Sex** | 2 | 1.43 | 0.243 | 0.014 | 2 | 0.77 | 0.462 |  |
| **Religion** | 1 | 0.76 | 0.384 | 0.004 | 1 | 1.22 | 0.271 |  |
| **Location (divisions)** | 8 | 1.02 | 0.422 | 0.04 | – | – | – |  |
| **Location (Major vs Minor)** | 1 | 3.11 | 0.08 | 0.016 | 1 | 1.87 | 0.173 |  |

R² = proportion of variance explained by each predictor for univariate. Dependent variable: pcl-5 total score.

# REFERENCES

1. Charan, J. and T. Biswas, *How to calculate sample size for different study designs in medical research?* Indian journal of psychological medicine, 2013. **35**(2): p. 121-126.

2. Pourhoseingholi, M.A., M. Vahedi, and M. Rahimzadeh, *Sample size calculation in medical studies.* Gastroenterology and Hepatology Bed Bench, 2013. **6**(1): p. 14-7.

3. Alam, M.F., et al., *Community prevalence of psychiatric disorders: Findings from a nationwide survey in Bangladesh.* Asian Journal of Psychiatry, 2024. **92**: p. 103897.

4. Ni, M.Y., et al., *Depression and post-traumatic stress during major social unrest in Hong Kong: a 10-year prospective cohort study.* The Lancet, 2020. **395**(10220): p. 273-284.

5. Bangladesh_Bureau_of-Statistics, *POPULATION & HOUSING CENSUS 2022*. 2022, Ministry of Planning: Bangladesh p. 68.

6. Statistics, B.B.o., *Population and Housing Census 2022*. 2023, Bangladesh Bureau of Statistics: Dhaka.

7. Jayalakshmi, T. and A. Santhakumaran, *Statistical normalization and back propagation for classification.* International Journal of Computer Theory and Engineering, 2011. **3**(1): p. 1793-8201.

8. Shantal, M., Z. Othman, and A.A. Bakar, *A novel approach for data feature weighting using correlation coefficients and min–max normalization.* Symmetry, 2023. **15**(12): p. 2185.

9. Fakhruddin, S., M. Babel, and A. Kawasaki, *Assessing the vulnerability of infrastructure to climate change on the Islands of Samoa.* Natural Hazards and Earth System Sciences, 2015. **15**(6): p. 1343-1356.
